# Clinical Lectures on Diseases of Women

**Published:** 1872-07

**Authors:** 


*Clinical Lectures on tke Diseases of Women.* By Sir James Y. Simpson, Bart., M. D. D. C. L. Edited by Alexander R. Simpson, M. D. New York: D. Appleton & Co. 1872.

The distinguished author of this work is too widely known in the department of Gynæcology to need any extended notice from us.

The present Volume comprises fifty clinical lectures delivered by Prof Simpson on Diseases of Women, ten of which have never appeared in print. The lectures are from notes taken by the Editor, Dr. Alexander R. Simpson, aided by the lecture notes of the author. Most of the lectures were published in the Medical Times and Gazette and passed under the immediate eye of the author. A copious Table of contents will materialy aid the reader, as the lectures being clinical are not arranged in a systematic order.

The Works of Prof. Simpson, of which this is the concluding Volume, should no other monument be erected to his memory, will serve to perpetuate his fame wherever the English language is spoken; enshrining in the minds of the Medical World the memory of a man, who by his patient and unceasing toil and valuable discoveries has done more than any man of his time in the cause of Gynæcology.

